# Activated STING: an ion channel to trigger non-interferon-related functions

**DOI:** 10.1038/s41392-023-01633-y

**Published:** 2023-10-16

**Authors:** Zhengyu Gao, Bin Wang, Long Zhang

**Affiliations:** 1 International Biomed-X Research Center, Second Affiliated Hospital of Zhejiang University School of Medicine, Zhejiang University, Hangzhou, China; 2 MOE Laboratory of Biosystems Homeostasis and Protection and Innovation Center for Cell Signaling Network, Life Sciences Institute, Zhejiang University, 310058 Hangzhou, China

**Keywords:** Cell biology, Biochemistry

Recently, a study published in *Science* by Liu et al.^1^ presented compelling evidence demonstrating that upon translocation to the Golgi apparatus, STING (Stimulator of Interferon Genes) serves as a direct mediator for proton release into the cytosol, thereby instigating non-canonical LC3B (light-chain 3B) lipidation and NLRP3 (NOD-like receptor family pyrin domain–containing 3) inflammasome activation (Fig. 1). This study offers a new perspective on the involvement of the cGAS-STING pathway in non-interferon-related functions.

**Fig. 1:**
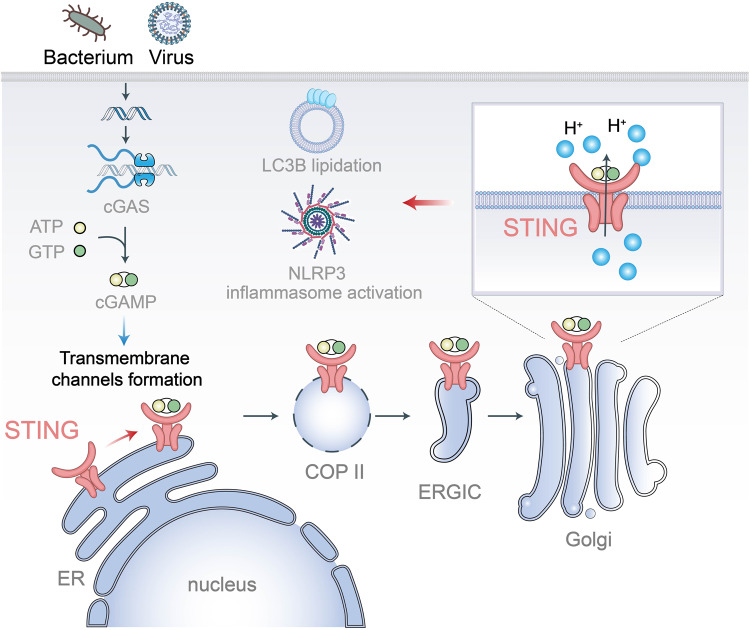
The functional model of activated STING as an ion channel. Upon activation, the STING protein translocates to the Golgi apparatus, forming a channel within the transmembrane region. This channel facilitates the efflux of protons, ultimately resulting in the induction of inflammasome activation and LC3B lipidation

cGAS (cyclic GMP-AMP synthase) is responsible for detecting cytosolic DNA, which indicates foreign entities, such as viruses, or damage to the host DNA. Upon binding to the cytosolic DNA, cGAS catalyzes the synthesis of cyclic nucleotides using ATP and GTP as substrates, activating STING.^2^ Active STING proteins undergo conformational alterations and relocate from the ER (endoplasmic reticulum) to the Golgi apparatus. Within these compartments, STING participates in multiple pathways, including interferon induction, non-canonical autophagy, and NLRP3 inflammasome activation. These notable discoveries have enriched our knowledge of the multifaceted roles of STING. In brief, the mechanism by which STING stimulates interferon production is well characterized. However, the exact mechanisms involved in the other two known non-interferon functions are still unclear and warrant further investigation.

Previous studies have demonstrated that cGAS-STING axis activates NLRP3 inflammasome independently of IFN induction^3^ and can trigger FIP200–independent non-canonical LC3B lipidation, thereby initiating “non-canonical autophagy”.^4,5^ Interestingly, proton leakage induced by the influenza virus M2 pore protein was found to be associated with non-canonical LC3B lipidation and NLRP3 inflammasome activation. In light of the established association between these processes and proton leakage in acidic organelles such as the Golgi and endosomes, researchers initially explored the potential involvement of organelle proton leakage in STING-induced LC3B lipidation and inflammasome activation. To assess this, they employed a Golgi-specific pH reporter (SEP-mRuby) that exhibits fluorescence in response to pH elevation. Through this approach, they have successfully observed that the activation of STING leads to an increase in pH within the Golgi apparatus. Notably, although the observed acidic organelle proton efflux is possibly linked to the initiation of non-canonical LC3B lipidation and NLRP3 inflammasome, no specific gene has been identified through genome-wide CRISPR screening to regulate this ion flow upon STING activation.

These findings prompted the authors to investigate whether proton efflux is mediated by activated STING rather than by other known transporters. To explore the structural domains of STING that may be involved in LC3B lipidation, the authors constructed oligomerization-deficient variants of STING and fusion proteins comprising the ligand-binding domain (LBD) of STING and endolysosomal-localized TMEM192 (TMEM192-STING-LBD). Following treatment with the STING agonist diABZI, an oligomerization-deficient variant of STING exhibits impaired STING translocation, phosphorylation, and LC3B lipidation. As for TMEM192-STING-LBD, despite its endolysosomal localization and a high degree of phosphorylation intensity, failed to induce LC3B lipidation, suggesting that translocation of the STING LBD alone is inadequate to initiate LC3B lipidation. Consequently, the research team postulated that the transmembrane domain of STING potentially plays a pivotal role in facilitating LC3B lipidation. Interestingly, the authors made a discovery through the structural analysis and comparison of STING’s cGAMP-bound (activated) and apo (inactivated) states. Active STING has pores that span the transmembrane region, whereas inactive STING features a central cavity that is confined within the membrane and does not extend across its entire span.

Serendipitously, while obtaining the channel prediction results, the team noticed a STING agonist, C53 (Compound 53), which was discovered last year, bound to the transmembrane region of STING. Realizing the potential of C53 to act as a channel blocker, the team seized the opportunity to test their hypotheses regarding the role of the STING ion channel. In cellular experiments, the addition of the STING activator diABZI or cGAMP increased Golgi pH. When diABZI and the potential channel blocker C53 were simultaneously administered, interferon activation was observed. However, the diABZI-induced increase in pH was significantly inhibited and the downstream pathways involved in autophagy initiation and inflammasome formation were not activated. Liu et al. achieved the first convincing experimental decoupling of these downstream processes by activating interferon production with C53 while inhibiting other two non-interferon-related pathways.

To further validate the involvement of STING in facilitating proton transport, purified STING proteins were reconstituted on liposomes for an in vitro liposome assay. The results demonstrated the occurrence of proton fluxes in liposomes incorporating STING, whereas the presence of C53 reduced these fluxes. In contrast, control liposomes did not exhibit any proton flux, suggesting that STING alone can transport protons.

In summary, the study conducted by Liu et al. proposed the existence of a proton channel within the transmembrane domain of the activated STING dimer. The channel facilitates proton transport, elevating Golgi pH, which triggers downstream non-interferon-related functions and can be effectively inhibited by specific channel inhibitors. Moreover, the researchers have successfully validated STING-mediated proton efflux through in vitro experiments using purified proteins reconstituted in liposomes. It is noteworthy that the authors identified the potential proton channel through the analysis of comparative chicken STING proteins. However, the functional validation of this channel was conducted in human cells, implying a conserved mechanism across species. Nevertheless, further structural and functional experiments are necessary to confirm this hypothesis. Moreover, the findings presented in this article hold promise for advancements in bacterial immunity research. STING proteins have been reported to originate from the prokaryotic immune system, where bacterial STING activates an effector domain that induces cell death as a defense mechanism against phage invasion upon recognition of a second messenger. Interestingly, certain transmembrane domains, which are presumed to disrupt the cell membrane, serve as effectors. This raises the question of whether bacterial STING forms channels within the transmembrane region, facilitating proton efflux upon recognition of the second messenger and ultimately triggering cell death. We believe that exploring this possibility holds significant scientific value. Anyway, given the significant implications of STING in host immunity, cellular senescence, and antitumor responses, there has been considerable interest in developing pharmacological agents capable of activating or inhibiting STING activity. The discovery of STING ion channel activity opens up new possibilities for designing therapeutic interventions that modulate STING function. These advances hold promise for the development of innovative treatment approaches.

## Data Availability

Not applicable.
